# 2-[2-(2,6-Dichloro­anilino)phen­yl]-*N*′-(4-propyl­cyclo­hexyl­idene)acetohydrazide

**DOI:** 10.1107/S1600536809054695

**Published:** 2010-01-09

**Authors:** Mehmet Akkurt, Mebble Nassozi, Gültaze Çapan, Ayşe Kocabalkanlı, Ísmail Çelik, Orhan Büyükgüngör

**Affiliations:** a Department of Physics, Faculty of Arts and Sciences, Erciyes University, 38039 Kayseri, Turkey; b Department of Pharmaceutical Chemistry, Faculty of Pharmacology, stanbul University, 34116 Beyazit, stanbul, Turkey; c Department of Physics, Faculty of Arts and Sciences, Cumhuriyet University, 58140 Sivas, Turkey; d Department of Physics, Faculty of Arts and Sciences, Ondokuz Mayıs University, 55139 Samsun, Turkey

## Abstract

The asymmetric unit of the title compound, C_23_H_27_Cl_2_N_3_O, contains two crystallographically independent mol­ecules in which the dihedral angles between the benzene rings are 70.1 (3) and 63.8 (3)°. In each mol­ecule an intra­molecular N—H⋯O hydrogen bond generates an *S*(7) ring. The atoms of the propyl grouping of one mol­ecule are disordered over two orientations with occupancies of 0.666 (6) and 0.334 (6). The crystal structure is stabilized by N—H⋯O and C—H⋯O hydrogen-bonding inter­actions.

## Related literature

For the pharmacological activity and biological properties of diclofenac and its derivatives, see: Gobec *et al.* (2005 ▶); Moser *et al.* (1990 ▶); Sallmann (1986 ▶); Sriram *et al.* (2006 ▶); Wittine *et al.* (2009 ▶); Zhang *et al.* (2009 ▶). For comparative bond lengths, *see*: Allen *et al.* (1987 ▶). For puckering parameters, *see*: Cremer & Pople (1975 ▶).
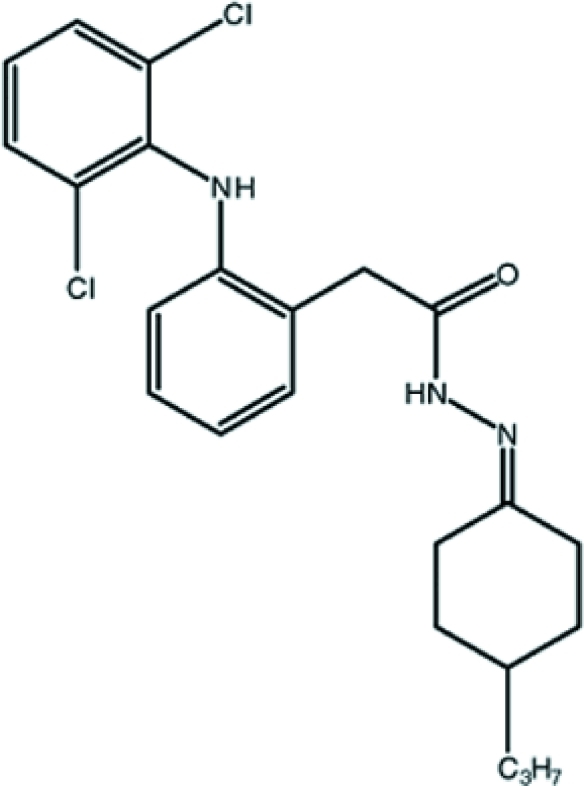


## Experimental

### 

#### Crystal data

C_23_H_27_Cl_2_N_3_O
*M* *_r_* = 432.38
Monoclinic, 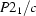

*a* = 13.0235 (6) Å
*b* = 15.2618 (5) Å
*c* = 26.6255 (12) Å
β = 118.098 (3)°
*V* = 4668.4 (4) Å^3^
*Z* = 8
Mo *K*α radiation
μ = 0.30 mm^−1^
*T* = 296 K
0.60 × 0.34 × 0.10 mm

#### Data collection

Stoe IPDS 2 diffractometer
Absorption correction: integration (*X-RED32*; Stoe & Cie, 2002 ▶) *T*_min_ = 0.842, *T*_max_ = 0.971
69169 measured reflections
9577 independent reflections
4744 reflections with *I* > 2σ(*I*)
*R*_int_ = 0.070

#### Refinement

*R*[*F* ^2^ > 2σ(*F* ^2^)] = 0.081
*wR*(*F* ^2^) = 0.247
*S* = 1.01
9577 reflections
492 parameters
7 restraints
H-atom parameters constrained
Δρ_max_ = 0.44 e Å^−3^
Δρ_min_ = −0.48 e Å^−3^

### 

Data collection: *X-AREA* (Stoe & Cie, 2002 ▶); cell refinement: *X-AREA*; data reduction: *X-RED32* (Stoe & Cie, 2002 ▶); program(s) used to solve structure: *SIR97* (Altomare *et al.*, 1999 ▶); program(s) used to refine structure: *SHELXL97* (Sheldrick, 2008 ▶); molecular graphics: *ORTEP-3 for Windows* (Farrugia, 1997 ▶); software used to prepare material for publication: *WinGX* (Farrugia, 1999 ▶).

## Supplementary Material

Crystal structure: contains datablocks global, I. DOI: 10.1107/S1600536809054695/hb5287sup1.cif

Structure factors: contains datablocks I. DOI: 10.1107/S1600536809054695/hb5287Isup2.hkl

Additional supplementary materials:  crystallographic information; 3D view; checkCIF report

## Figures and Tables

**Table 1:** Hydrogen-bond geometry (Å, °)

*D*—H⋯*A*	*D*—H	H⋯*A*	*D*⋯*A*	*D*—H⋯*A*
N1—H1⋯O1	0.86	2.28	2.886 (4)	128
N2—H2⋯O2^i^	0.86	2.24	3.077 (5)	165
N4—H4*A*⋯O2	0.86	2.32	2.921 (6)	127
N5—H5*A*⋯O1^ii^	0.86	2.21	3.034 (4)	161
C20—H20*A*⋯O2^i^	0.97	2.53	3.330 (6)	139
C36—H36*B*⋯O1^ii^	0.97	2.41	3.307 (5)	155

Symmetry codes: (i) 
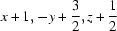
; (ii) 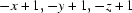.

**Fig. 1.**
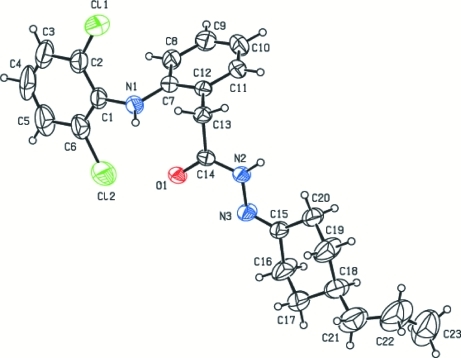
View of the non-disorder molecule of (I) with displacement ellipsoids for non-H atoms drawn at the 20% probability level.

**Fig. 2.**
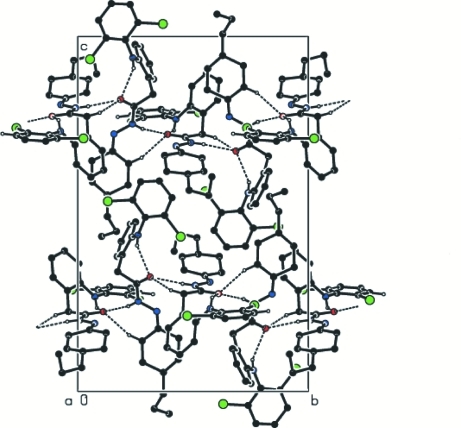
View of the packing and hydrogen bonding interactions of (I) down the a axis. All hydrogen atoms not involved in hydrogen bonding have been omitted for clarity.

## supplementary crystallographic information

### Comment

Diclofenac, [2-(2,6-dichlorophenylamino)phenyl]acetic acid, has long been widely used for its antipyretic, analgesic, and antiinflammatory activities (Moser *et al.*, 1990; Sallmann, 1986). Besides, diclofenac and its derivatives displayed various biological properties like anticancer (Gobec *et al.*, 2005), antimycobacterial (Sriram *et al.*, 2006), antiviral (Wittine *et al.*, 2009) and insulin-sensitizing activity (Zhang *et al.*, 2009).

In the title compound (I), the asymmetric unit contains two crystallographically independent molecules. Fig. 1 shows the non-disorder molecule. In the molecules A and B, bond lengthsdistances are within the expected range (Allen *et al.*, 1987) and the dihedral angles between the benzene rings are 70.1 (3)° and 63.8 (3)°, respectively. Molecules A and B have a distorted chair conformation with the puckering parameters [Cremer & Pople (1975)]; Q_T_ = 0.536 (7) Å, θ = 169.8 (7)°, φ = 350 (5)° and Q_T_ = 0.363 (10) Å, θ = 23.4 (14)°, φ = 232 (4)°, respectively. The crystal structure is stabilized by N—H···O, C—H···O and C—H···N hydrogen bonding interactions (Table 1 and Fig. 2).

### Experimental

A mixture of 2-{2-[(2,6-dichlorophenyl)amino]phenyl}acetohydrazide (0.005 mol) and 4-propylcyclohexanone (0.01 mol) was refluxed in 15 ml *ABS*. EtOH for 5 h. The precipitate obtained was filtered, dried and purified by recrystallization from EtOH. Yield, 71.8%, m.p. 474.2 - 474.8 K. UV (EtOH) l max = 281.6, 205.0 nm. IR (KBr) n = 3226 (N—H), 1651 (C=O, C=N) cm^-1^. ^1^H-NMR (DMSO-d_6_, 500 MHz) d = 0.91- 0.95 (3*H*, m, CH_3_-cyc*.), 1.04–1.16 (1*H*, m, CH_2_-cyc.), 1.22–1.29,1.32–1.40 (4*H*, 2 m, CH_2_CH_2_CH_3_-cyc.), 1.57–1.62 (1*H*, m, CH-cyc.), 1.88–2.01 (3*H*, m, CH_2_-cyc.), 2.21–2.29 (1*H*, m, CH_2_-cyc.), 2.38–2.42 (1*H*, m, CH_2_-cyc.), 2.95–3.03(1*H*, m, CH_2_-cyc.), 3.76, 4.03, 4.07 (2*H*, s, 2 d, J = 13.6 Hz, CH_2_CO), 6.30, 6.36 (1*H*, 2 d, J = 7.81 Hz, Ar—H*), 6.88, 6.92 (1*H*, 2 t, J = 7.32 Hz, Ar—H), 7.08–7.12 (1*H*, m, Ar—H), 7.21–7.32 (2*H*, m, Ar—H), 7.57–7.59 (2*H*, m, Ar—H), 7.87,8.35 (1*H*, 2 s, NH), 10.60, 10.62 (1*H*, 2 s, CONH) p.p.m. (*cyc.= cyclohexylidene, Ar= aromatic). Analysis calculated for C_23_H_27_Cl_2_N_3_O: C 63.89, H6.25, N 9.72%. Found: C63.23, H 6.37, N 9.61%.

### Refinement

H atoms were positioned geometrically with N—H = 0.86 Å, C—H = 0.93 - 0.97 Å and refined using a riding model with *U*_iso_(H) = 1.2 or 1.5*U*_eq_(C, N). The atoms of the propyl moiety of the disorder molecule are disordered over two positions with the site occupation factors 0.666 (6) and 0.334 (6).

### Crystal data

**Table:**

C_23_H_27_Cl_2_N_3_O	*F*(000) = 1824
*M**_r_* = 432.38	*D*_x_ = 1.230 Mg m^−^^3^
Monoclinic, *P*2_1_/*c*	Mo *K*α radiation, λ = 0.71073 Å
Hall symbol: -P 2ybc	Cell parameters from 39814 reflections
*a* = 13.0235 (6) Å	θ = 1.6–27.2°
*b* = 15.2618 (5) Å	µ = 0.30 mm^−^^1^
*c* = 26.6255 (12) Å	*T* = 296 K
β = 118.098 (3)°	Prism, colourless
*V* = 4668.4 (4) Å^3^	0.60 × 0.34 × 0.10 mm
*Z* = 8	

### Data collection

**Table:**

Stoe IPDS 2 diffractometer	9577 independent reflections
Radiation source: sealed X-ray tube, 12 x 0.4 mm long-fine focus	4744 reflections with *I* > 2σ(*I*)
plane graphite	*R*_int_ = 0.070
Detector resolution: 6.67 pixels mm^-1^	θ_max_ = 26.5°, θ_min_ = 1.7°
ω scans	*h* = −16→16
Absorption correction: integration (*X-RED3*; Stoe & Cie, 2002)	*k* = −19→19
*T*_min_ = 0.842, *T*_max_ = 0.971	*l* = −33→33
69169 measured reflections	

### Refinement

**Table:**

Refinement on *F*^2^	Primary atom site location: structure-invariant direct methods
Least-squares matrix: full	Secondary atom site location: difference Fourier map
*R*[*F*^2^ > 2σ(*F*^2^)] = 0.081	Hydrogen site location: inferred from neighbouring sites
*wR*(*F*^2^) = 0.247	H-atom parameters constrained
*S* = 1.01	*w* = 1/[σ^2^(*F*_o_^2^) + (0.1324*P*)^2^] where *P* = (*F*_o_^2^ + 2*F*_c_^2^)/3
9577 reflections	(Δ/σ)_max_ < 0.001
492 parameters	Δρ_max_ = 0.44 e Å^−^^3^
7 restraints	Δρ_min_ = −0.48 e Å^−^^3^

### Special details

**Table:**

Geometry. Bond distances, angles *etc*. have been calculated using the rounded fractional coordinates. All su's are estimated from the variances of the (full) variance-covariance matrix. The cell e.s.d.'s are taken into account in the estimation of distances, angles and torsion angles
Refinement. Refinement on *F*^2^ for ALL reflections except those flagged by the user for potential systematic errors. Weighted *R*-factors *wR* and all goodnesses of fit *S* are based on *F*^2^, conventional *R*-factors *R* are based on *F*, with *F* set to zero for negative *F*^2^. The observed criterion of *F*^2^ > σ(*F*^2^) is used only for calculating -*R*-factor-obs *etc*. and is not relevant to the choice of reflections for refinement. *R*-factors based on *F*^2^ are statistically about twice as large as those based on *F*, and *R*-factors based on ALL data will be even larger.

### Fractional atomic coordinates and isotropic or equivalent isotropic displacement parameters (Å^2^)

**Table:**

	*x*	*y*	*z*	*U*_iso_*/*U*_eq_	Occ. (<1)
Cl3	0.55339 (12)	0.47497 (7)	0.21299 (7)	0.0961 (5)	
Cl4	0.35062 (13)	0.76589 (7)	0.24245 (6)	0.0961 (5)	
O2	0.2070 (3)	0.61054 (15)	0.27547 (13)	0.0687 (10)	
N4	0.3728 (3)	0.57460 (19)	0.23307 (14)	0.0629 (11)	
N5	0.1122 (3)	0.50946 (18)	0.29889 (14)	0.0619 (11)	
N6	0.0476 (3)	0.5731 (2)	0.30818 (16)	0.0723 (13)	
C24	0.4513 (3)	0.6241 (2)	0.22301 (16)	0.0591 (11)	
C25	0.4538 (4)	0.7154 (2)	0.22791 (19)	0.0714 (16)	
C26	0.5337 (5)	0.7657 (3)	0.2219 (2)	0.097 (2)	
C27	0.6145 (5)	0.7280 (3)	0.2095 (3)	0.100 (2)	
C28	0.6163 (4)	0.6386 (3)	0.2052 (2)	0.0875 (19)	
C29	0.5386 (4)	0.5878 (3)	0.21322 (19)	0.0714 (14)	
C30	0.2970 (3)	0.5115 (2)	0.19509 (19)	0.0624 (13)	
C31	0.2774 (4)	0.5052 (3)	0.1398 (2)	0.0832 (17)	
C32	0.1971 (5)	0.4428 (5)	0.1038 (3)	0.123 (3)	
C33	0.1421 (6)	0.3871 (5)	0.1238 (4)	0.141 (4)	
C34	0.1628 (5)	0.3935 (3)	0.1788 (3)	0.103 (3)	
C35	0.2404 (4)	0.4557 (2)	0.2156 (2)	0.0680 (15)	
C36	0.2595 (4)	0.4600 (2)	0.2751 (2)	0.0689 (15)	
C37	0.1899 (3)	0.5338 (2)	0.28315 (16)	0.0544 (11)	
C38	−0.0211 (5)	0.5513 (3)	0.3267 (3)	0.091 (2)	
C39	−0.0417 (7)	0.4612 (4)	0.3418 (4)	0.1403 (18)	
C40	−0.0837 (7)	0.4531 (4)	0.3797 (4)	0.1403 (18)	
C41	−0.1573 (8)	0.5226 (4)	0.3871 (4)	0.1403 (18)	
C42	−0.1138 (7)	0.6138 (4)	0.3802 (4)	0.1403 (18)	
C43	−0.0909 (7)	0.6232 (4)	0.3349 (4)	0.1403 (18)	
C44A	−0.1851 (13)	0.5169 (6)	0.4331 (7)	0.1405 (3)	0.666 (6)
C45A	−0.2286 (11)	0.4326 (6)	0.4477 (6)	0.1405 (3)	0.666 (6)
C46A	−0.3406 (11)	0.4181 (7)	0.3950 (6)	0.1405 (3)	0.666 (6)
C46B	−0.337 (2)	0.3799 (11)	0.4379 (11)	0.1405 (3)	0.334 (6)
C44B	−0.276 (2)	0.4895 (11)	0.3812 (13)	0.1405 (3)	0.334 (6)
C45B	−0.274 (3)	0.3976 (12)	0.4063 (13)	0.1405 (3)	0.334 (6)
Cl1	0.65421 (16)	0.86931 (15)	0.46429 (9)	0.1470 (9)	
Cl2	0.69658 (17)	0.55357 (12)	0.56388 (8)	0.1386 (8)	
O1	0.8618 (2)	0.68597 (16)	0.67741 (11)	0.0660 (9)	
N1	0.7624 (3)	0.7387 (2)	0.55967 (14)	0.0720 (12)	
N2	1.0330 (3)	0.73731 (17)	0.74608 (13)	0.0600 (10)	
N3	1.0550 (3)	0.65711 (19)	0.77439 (16)	0.0744 (11)	
C1	0.6732 (4)	0.7070 (4)	0.50919 (19)	0.0790 (18)	
C2	0.6183 (4)	0.7609 (4)	0.4604 (2)	0.098 (2)	
C3	0.5347 (6)	0.7271 (7)	0.4093 (3)	0.132 (4)	
C4	0.4974 (6)	0.6461 (9)	0.4068 (4)	0.152 (4)	
C5	0.5440 (6)	0.5910 (6)	0.4539 (4)	0.143 (3)	
C6	0.6327 (5)	0.6232 (4)	0.5040 (2)	0.102 (2)	
C7	0.8708 (3)	0.7650 (3)	0.56500 (17)	0.0646 (12)	
C8	0.9009 (4)	0.7488 (3)	0.52235 (19)	0.0817 (19)	
C9	1.0070 (5)	0.7718 (4)	0.5287 (2)	0.0962 (19)	
C10	1.0875 (5)	0.8114 (4)	0.5773 (3)	0.104 (2)	
C11	1.0586 (4)	0.8288 (3)	0.6199 (2)	0.0831 (17)	
C12	0.9510 (3)	0.8072 (2)	0.61494 (16)	0.0616 (12)	
C13	0.9229 (4)	0.8264 (2)	0.66274 (17)	0.0633 (11)	
C14	0.9359 (3)	0.7440 (2)	0.69641 (16)	0.0541 (11)	
C15	1.1410 (4)	0.6509 (3)	0.82318 (19)	0.0729 (16)	
C16	1.1645 (6)	0.5615 (3)	0.8499 (3)	0.129 (3)	
C17	1.1834 (6)	0.5634 (4)	0.9097 (3)	0.136 (3)	
C18	1.2791 (6)	0.6281 (4)	0.9464 (2)	0.105 (3)	
C19	1.2429 (7)	0.7183 (4)	0.9183 (3)	0.136 (3)	
C20	1.2220 (5)	0.7205 (4)	0.8578 (2)	0.117 (2)	
C21	1.3058 (8)	0.6285 (7)	1.0070 (3)	0.161 (4)	
C22	1.4223 (12)	0.6681 (8)	1.0492 (4)	0.211 (6)	
C23	1.5268 (10)	0.6037 (11)	1.0633 (4)	0.233 (8)	
H5A	0.10180	0.45490	0.30330	0.0740*	
H28	0.67070	0.61230	0.19670	0.1050*	
H26	0.53340	0.82620	0.22630	0.1170*	
H27	0.66730	0.76250	0.20410	0.1190*	
H4A	0.37050	0.58310	0.26450	0.0760*	
H34	0.12460	0.35600	0.19200	0.1240*	
H36A	0.34170	0.46900	0.30060	0.0830*	
H36B	0.23700	0.40470	0.28500	0.0830*	
H39A	−0.09560	0.43200	0.30690	0.1690*	
H31	0.31660	0.54160	0.12650	0.1000*	
H32	0.18110	0.43920	0.06600	0.1470*	
H33	0.09070	0.34490	0.09990	0.1690*	
H41	−0.23290	0.51660	0.35300	0.1690*	
H42A	−0.17180	0.65690	0.37630	0.1690*	
H42B	−0.04330	0.62730	0.41490	0.1690*	
H43A	−0.04980	0.67800	0.33960	0.1690*	
H43B	−0.16470	0.62790	0.30040	0.1690*	
H44A	−0.24360	0.56130	0.42620	0.1690*	0.666 (6)
H44B	−0.11580	0.53440	0.46730	0.1690*	0.666 (6)
H45A	−0.17480	0.38450	0.45460	0.1690*	0.666 (6)
H45B	−0.24070	0.44020	0.48070	0.1690*	0.666 (6)
H46A	−0.37430	0.36360	0.39800	0.2110*	0.666 (6)
H46B	−0.32670	0.41600	0.36270	0.2110*	0.666 (6)
H46C	−0.39320	0.46520	0.39040	0.2110*	0.666 (6)
H39B	0.03140	0.42940	0.35690	0.1690*	
H40A	−0.01700	0.44400	0.41680	0.1690*	
H40B	−0.12860	0.39930	0.37020	0.1690*	
H44C	−0.30320	0.53140	0.39980	0.1690*	0.334 (6)
H44D	−0.33270	0.48880	0.34110	0.1690*	0.334 (6)
H45C	−0.30180	0.35630	0.37490	0.1690*	0.334 (6)
H45D	−0.19290	0.38330	0.43120	0.1690*	0.334 (6)
H46D	−0.36230	0.31990	0.43240	0.2110*	0.334 (6)
H46E	−0.40380	0.41780	0.42450	0.2110*	0.334 (6)
H46F	−0.28720	0.39040	0.47770	0.2110*	0.334 (6)
H1	0.75110	0.74250	0.58890	0.0870*	
H2	1.08010	0.78080	0.76000	0.0720*	
H3	0.50470	0.76150	0.37660	0.1590*	
H4	0.43830	0.62520	0.37250	0.1820*	
H5	0.51620	0.53430	0.45180	0.1720*	
H8	0.84750	0.72170	0.48890	0.0980*	
H9	1.02510	0.76040	0.49950	0.1150*	
H10	1.16040	0.82640	0.58150	0.1240*	
H11	1.11320	0.85590	0.65310	0.1000*	
H13A	0.97520	0.87120	0.68750	0.0760*	
H13B	0.84380	0.84820	0.64720	0.0760*	
H16A	1.09920	0.52350	0.82740	0.1560*	
H16B	1.23290	0.53710	0.84950	0.1560*	
H17A	1.20460	0.50530	0.92600	0.1630*	
H17B	1.11140	0.57990	0.90960	0.1630*	
H18	1.35020	0.61090	0.94500	0.1260*	
H19A	1.17240	0.73640	0.91910	0.1630*	
H19B	1.30340	0.76030	0.94040	0.1630*	
H20A	1.19010	0.77710	0.84130	0.1400*	
H20B	1.29550	0.71330	0.85740	0.1400*	
H21A	1.30320	0.56860	1.01840	0.1930*	
H21B	1.24450	0.66050	1.01000	0.1930*	
H22A	1.42000	0.68270	1.08410	0.2540*	
H22B	1.43490	0.72190	1.03350	0.2540*	
H23A	1.54970	0.57660	1.09960	0.3460*	
H23B	1.59130	0.63580	1.06450	0.3460*	
H23C	1.50300	0.55940	1.03440	0.3460*	

### Atomic displacement parameters (Å^2^)

**Table:**

	*U*^11^	*U*^22^	*U*^33^	*U*^12^	*U*^13^	*U*^23^
Cl3	0.0978 (9)	0.0652 (6)	0.1428 (12)	0.0112 (6)	0.0711 (9)	0.0132 (7)
Cl4	0.1193 (10)	0.0505 (5)	0.1282 (11)	−0.0088 (6)	0.0662 (9)	−0.0128 (6)
O2	0.096 (2)	0.0376 (12)	0.093 (2)	−0.0001 (12)	0.0614 (17)	0.0008 (12)
N4	0.080 (2)	0.0532 (16)	0.063 (2)	−0.0136 (15)	0.0398 (17)	−0.0040 (14)
N5	0.078 (2)	0.0378 (14)	0.081 (2)	0.0039 (14)	0.0467 (19)	0.0030 (14)
N6	0.091 (2)	0.0505 (17)	0.094 (3)	0.0165 (16)	0.059 (2)	0.0134 (16)
C24	0.065 (2)	0.0496 (19)	0.057 (2)	−0.0087 (16)	0.0240 (19)	0.0042 (16)
C25	0.083 (3)	0.051 (2)	0.076 (3)	−0.0135 (19)	0.034 (2)	0.0001 (18)
C26	0.113 (4)	0.063 (3)	0.117 (4)	−0.032 (3)	0.055 (3)	−0.003 (3)
C27	0.094 (4)	0.080 (3)	0.125 (5)	−0.031 (3)	0.052 (3)	0.007 (3)
C28	0.074 (3)	0.095 (3)	0.095 (4)	−0.009 (2)	0.041 (3)	0.011 (3)
C29	0.069 (2)	0.064 (2)	0.078 (3)	−0.005 (2)	0.032 (2)	0.010 (2)
C30	0.068 (2)	0.0468 (18)	0.079 (3)	−0.0026 (17)	0.040 (2)	−0.0128 (17)
C31	0.079 (3)	0.097 (3)	0.083 (3)	−0.008 (2)	0.046 (3)	−0.024 (3)
C32	0.103 (4)	0.167 (6)	0.107 (5)	−0.020 (4)	0.057 (4)	−0.079 (4)
C33	0.099 (4)	0.173 (7)	0.177 (7)	−0.067 (4)	0.087 (5)	−0.110 (6)
C34	0.098 (4)	0.082 (3)	0.164 (6)	−0.034 (3)	0.090 (4)	−0.059 (3)
C35	0.076 (3)	0.0403 (18)	0.105 (3)	−0.0046 (17)	0.057 (2)	−0.0143 (19)
C36	0.083 (3)	0.0376 (17)	0.107 (3)	0.0062 (17)	0.062 (3)	0.0117 (19)
C37	0.068 (2)	0.0371 (16)	0.061 (2)	0.0003 (15)	0.0329 (19)	0.0028 (15)
C38	0.105 (4)	0.065 (3)	0.135 (5)	0.020 (2)	0.083 (4)	0.017 (3)
C39	0.189 (3)	0.0902 (17)	0.221 (4)	0.0279 (19)	0.162 (3)	0.013 (2)
C40	0.189 (3)	0.0902 (17)	0.221 (4)	0.0279 (19)	0.162 (3)	0.013 (2)
C41	0.189 (3)	0.0902 (17)	0.221 (4)	0.0279 (19)	0.162 (3)	0.013 (2)
C42	0.189 (3)	0.0902 (17)	0.221 (4)	0.0279 (19)	0.162 (3)	0.013 (2)
C43	0.189 (3)	0.0902 (17)	0.221 (4)	0.0279 (19)	0.162 (3)	0.013 (2)
C44A	0.1887 (7)	0.0902 (4)	0.2215 (4)	0.0279 (4)	0.1617 (4)	0.0134 (7)
C45A	0.1887 (7)	0.0902 (4)	0.2215 (4)	0.0279 (4)	0.1617 (4)	0.0134 (7)
C46A	0.1887 (7)	0.0902 (4)	0.2215 (4)	0.0279 (4)	0.1617 (4)	0.0134 (7)
C46B	0.1887 (7)	0.0902 (4)	0.2215 (4)	0.0279 (4)	0.1617 (4)	0.0134 (7)
C44B	0.1887 (7)	0.0902 (4)	0.2215 (4)	0.0279 (4)	0.1617 (4)	0.0134 (7)
C45B	0.1887 (7)	0.0902 (4)	0.2215 (4)	0.0279 (4)	0.1617 (4)	0.0134 (7)
Cl1	0.1150 (12)	0.1718 (17)	0.1371 (15)	0.0079 (11)	0.0452 (11)	0.0732 (13)
Cl2	0.1478 (15)	0.1123 (11)	0.1266 (14)	−0.0535 (11)	0.0407 (11)	−0.0050 (9)
O1	0.0732 (17)	0.0547 (14)	0.0643 (17)	−0.0151 (12)	0.0276 (14)	0.0023 (12)
N1	0.066 (2)	0.093 (2)	0.056 (2)	−0.0175 (18)	0.0280 (17)	−0.0021 (17)
N2	0.0691 (19)	0.0391 (14)	0.063 (2)	−0.0077 (13)	0.0239 (17)	0.0032 (13)
N3	0.085 (2)	0.0406 (15)	0.076 (2)	−0.0033 (15)	0.020 (2)	0.0104 (15)
C1	0.056 (2)	0.117 (4)	0.058 (3)	−0.014 (2)	0.022 (2)	−0.006 (2)
C2	0.059 (3)	0.159 (5)	0.072 (3)	−0.006 (3)	0.027 (3)	0.004 (3)
C3	0.067 (4)	0.229 (9)	0.075 (4)	−0.003 (5)	0.012 (3)	0.001 (5)
C4	0.065 (4)	0.262 (11)	0.089 (5)	−0.022 (6)	0.003 (3)	−0.054 (7)
C5	0.094 (4)	0.183 (7)	0.126 (6)	−0.049 (5)	0.030 (5)	−0.047 (6)
C6	0.081 (3)	0.133 (5)	0.083 (4)	−0.035 (3)	0.031 (3)	−0.026 (3)
C7	0.060 (2)	0.072 (2)	0.060 (2)	−0.0043 (19)	0.0268 (19)	0.0109 (19)
C8	0.071 (3)	0.112 (4)	0.057 (3)	−0.012 (2)	0.026 (2)	0.002 (2)
C9	0.083 (3)	0.135 (4)	0.079 (3)	0.001 (3)	0.045 (3)	0.019 (3)
C10	0.075 (3)	0.146 (5)	0.092 (4)	−0.030 (3)	0.041 (3)	0.009 (3)
C11	0.072 (3)	0.096 (3)	0.069 (3)	−0.027 (2)	0.023 (2)	0.008 (2)
C12	0.064 (2)	0.057 (2)	0.056 (2)	−0.0109 (17)	0.0219 (19)	0.0078 (17)
C13	0.073 (2)	0.0442 (18)	0.064 (2)	−0.0032 (16)	0.025 (2)	0.0032 (16)
C14	0.063 (2)	0.0405 (17)	0.061 (2)	−0.0035 (15)	0.031 (2)	−0.0010 (15)
C15	0.075 (3)	0.054 (2)	0.072 (3)	−0.0043 (19)	0.020 (2)	0.0141 (19)
C16	0.153 (6)	0.064 (3)	0.102 (5)	0.002 (3)	0.003 (4)	0.030 (3)
C17	0.109 (4)	0.119 (5)	0.128 (6)	−0.017 (4)	0.014 (4)	0.058 (4)
C18	0.125 (5)	0.098 (4)	0.086 (4)	0.006 (3)	0.044 (3)	0.024 (3)
C19	0.182 (7)	0.097 (4)	0.075 (4)	−0.002 (4)	0.015 (4)	−0.001 (3)
C20	0.121 (4)	0.095 (4)	0.077 (4)	−0.043 (3)	0.000 (3)	0.026 (3)
C21	0.168 (8)	0.203 (9)	0.108 (6)	0.030 (7)	0.063 (6)	0.044 (6)
C22	0.251 (13)	0.250 (13)	0.075 (5)	−0.011 (11)	0.029 (7)	−0.008 (6)
C23	0.150 (8)	0.41 (2)	0.126 (8)	0.031 (11)	0.055 (6)	0.020 (10)

### Geometric parameters (Å, °)

**Table:**

Cl3—C29	1.733 (5)	C44A—H44A	0.9700
Cl4—C25	1.743 (6)	C44A—H44B	0.9700
Cl1—C2	1.709 (6)	C44B—H44C	0.9700
Cl2—C6	1.765 (6)	C44B—H44D	0.9700
O2—C37	1.227 (4)	C44B—H41	1.2000
O1—C14	1.230 (5)	C45A—H45B	0.9700
N4—C24	1.395 (6)	C45A—H45A	0.9700
N4—C30	1.409 (5)	C45B—H45C	0.9700
N5—N6	1.381 (5)	C45B—H45D	0.9700
N5—C37	1.318 (6)	C46A—H46C	0.9600
N6—C38	1.253 (8)	C46A—H46A	0.9600
N4—H4A	0.8600	C46A—H46B	0.9600
N5—H5A	0.8600	C46B—H46E	0.9600
N1—C1	1.385 (6)	C46B—H46F	0.9600
N1—C7	1.409 (6)	C46B—H46D	0.9600
N2—N3	1.395 (4)	C1—C2	1.414 (7)
N2—C14	1.335 (5)	C1—C6	1.365 (9)
N3—C15	1.257 (6)	C2—C3	1.381 (9)
N1—H1	0.8600	C3—C4	1.318 (17)
N2—H2	0.8600	C4—C5	1.390 (15)
C24—C29	1.395 (7)	C5—C6	1.380 (11)
C24—C25	1.398 (4)	C7—C12	1.402 (6)
C25—C26	1.362 (8)	C7—C8	1.387 (7)
C26—C27	1.369 (10)	C8—C9	1.357 (9)
C27—C28	1.370 (7)	C9—C10	1.364 (9)
C28—C29	1.369 (8)	C10—C11	1.379 (9)
C30—C31	1.374 (7)	C11—C12	1.384 (7)
C30—C35	1.395 (6)	C12—C13	1.509 (6)
C31—C32	1.405 (9)	C13—C14	1.507 (5)
C32—C33	1.371 (11)	C15—C20	1.475 (8)
C33—C34	1.362 (12)	C15—C16	1.502 (7)
C34—C35	1.396 (7)	C16—C17	1.492 (10)
C35—C36	1.485 (7)	C17—C18	1.528 (10)
C36—C37	1.523 (6)	C18—C21	1.482 (9)
C38—C43	1.505 (10)	C18—C19	1.531 (9)
C38—C39	1.492 (9)	C19—C20	1.502 (9)
C39—C40	1.361 (14)	C21—C22	1.524 (16)
C40—C41	1.504 (13)	C22—C23	1.57 (2)
C41—C44B	1.56 (3)	C3—H3	0.9300
C41—C42	1.546 (10)	C4—H4	0.9300
C41—C44A	1.43 (2)	C5—H5	0.9300
C42—C43	1.380 (14)	C8—H8	0.9300
C44A—C45A	1.528 (17)	C9—H9	0.9300
C44B—C45B	1.55 (3)	C10—H10	0.9300
C45A—C46A	1.49 (2)	C11—H11	0.9300
C45B—C46B	1.45 (5)	C13—H13A	0.9700
C26—H26	0.9300	C13—H13B	0.9700
C27—H27	0.9300	C16—H16A	0.9700
C28—H28	0.9300	C16—H16B	0.9700
C31—H31	0.9300	C17—H17A	0.9700
C32—H32	0.9300	C17—H17B	0.9700
C33—H33	0.9300	C18—H18	0.9800
C34—H34	0.9300	C19—H19A	0.9700
C36—H36B	0.9700	C19—H19B	0.9700
C36—H36A	0.9700	C20—H20A	0.9700
C39—H39B	0.9700	C20—H20B	0.9700
C39—H39A	0.9700	C21—H21A	0.9700
C40—H40B	0.9700	C21—H21B	0.9700
C40—H40A	0.9700	C22—H22A	0.9700
C41—H41	0.9800	C22—H22B	0.9700
C42—H42B	0.9700	C23—H23A	0.9600
C42—H42A	0.9700	C23—H23B	0.9600
C43—H43A	0.9700	C23—H23C	0.9600
C43—H43B	0.9700		
			
C24—N4—C30	124.2 (3)	C44A—C45A—H45B	112.00
N6—N5—C37	118.8 (3)	C44A—C45A—H45A	111.00
N5—N6—C38	119.5 (4)	C44B—C45B—H45C	107.00
C24—N4—H4A	118.00	C44B—C45B—H45D	107.00
C30—N4—H4A	118.00	C46B—C45B—H45C	107.00
C37—N5—H5A	121.00	C46B—C45B—H45D	107.00
N6—N5—H5A	121.00	H45C—C45B—H45D	107.00
C1—N1—C7	122.7 (4)	C45A—C46A—H46A	109.00
N3—N2—C14	117.4 (3)	H46A—C46A—H46B	109.00
N2—N3—C15	118.8 (4)	H46B—C46A—H46C	110.00
C7—N1—H1	119.00	C45A—C46A—H46C	109.00
C1—N1—H1	119.00	H46A—C46A—H46C	109.00
N3—N2—H2	121.00	C45A—C46A—H46B	110.00
C14—N2—H2	121.00	C45B—C46B—H46D	110.00
N4—C24—C25	120.6 (4)	H46E—C46B—H46F	109.00
N4—C24—C29	123.8 (3)	C45B—C46B—H46E	109.00
C25—C24—C29	115.3 (4)	C45B—C46B—H46F	110.00
Cl4—C25—C26	119.2 (3)	H46D—C46B—H46E	109.00
C24—C25—C26	122.4 (5)	H46D—C46B—H46F	109.00
Cl4—C25—C24	118.3 (4)	N1—C1—C2	121.2 (5)
C25—C26—C27	120.5 (4)	N1—C1—C6	122.2 (4)
C26—C27—C28	118.9 (6)	C2—C1—C6	116.6 (5)
C27—C28—C29	120.5 (5)	Cl1—C2—C3	119.2 (5)
Cl3—C29—C28	118.1 (4)	C1—C2—C3	120.7 (6)
C24—C29—C28	122.1 (4)	Cl1—C2—C1	120.1 (4)
Cl3—C29—C24	119.8 (4)	C2—C3—C4	120.1 (8)
N4—C30—C35	117.2 (4)	C3—C4—C5	121.9 (9)
N4—C30—C31	122.1 (4)	C4—C5—C6	117.9 (8)
C31—C30—C35	120.7 (4)	Cl2—C6—C5	118.9 (6)
C30—C31—C32	118.8 (5)	C1—C6—C5	122.6 (6)
C31—C32—C33	120.8 (7)	Cl2—C6—C1	118.5 (4)
C32—C33—C34	119.9 (7)	N1—C7—C12	119.3 (4)
C33—C34—C35	121.0 (6)	C8—C7—C12	119.1 (4)
C30—C35—C36	122.5 (4)	N1—C7—C8	121.5 (4)
C30—C35—C34	118.8 (5)	C7—C8—C9	121.1 (4)
C34—C35—C36	118.7 (5)	C8—C9—C10	121.0 (6)
C35—C36—C37	111.8 (3)	C9—C10—C11	118.7 (6)
O2—C37—N5	123.3 (4)	C10—C11—C12	122.2 (5)
N5—C37—C36	115.7 (3)	C7—C12—C13	121.4 (4)
O2—C37—C36	121.0 (4)	C11—C12—C13	120.7 (4)
N6—C38—C39	127.1 (6)	C7—C12—C11	117.9 (4)
N6—C38—C43	117.1 (5)	C12—C13—C14	109.6 (3)
C39—C38—C43	115.8 (7)	N2—C14—C13	116.2 (3)
C38—C39—C40	118.0 (6)	O1—C14—N2	123.1 (3)
C39—C40—C41	122.5 (7)	O1—C14—C13	120.7 (4)
C42—C41—C44A	114.2 (7)	N3—C15—C20	128.2 (4)
C42—C41—C44B	132.8 (10)	C16—C15—C20	115.6 (5)
C40—C41—C42	109.1 (8)	N3—C15—C16	116.2 (5)
C40—C41—C44A	120.1 (8)	C15—C16—C17	112.5 (5)
C40—C41—C44B	114.9 (9)	C16—C17—C18	111.7 (6)
C41—C42—C43	116.1 (7)	C17—C18—C19	107.3 (5)
C38—C43—C42	116.4 (6)	C17—C18—C21	113.7 (7)
C41—C44A—C45A	122.8 (10)	C19—C18—C21	112.9 (6)
C41—C44B—C45B	116 (2)	C18—C19—C20	113.7 (5)
C44A—C45A—C46A	101.6 (11)	C15—C20—C19	111.7 (5)
C44B—C45B—C46B	121 (2)	C18—C21—C22	116.5 (9)
C25—C26—H26	120.00	C21—C22—C23	112.1 (10)
C27—C26—H26	120.00	C2—C3—H3	120.00
C28—C27—H27	121.00	C4—C3—H3	120.00
C26—C27—H27	121.00	C3—C4—H4	119.00
C29—C28—H28	120.00	C5—C4—H4	119.00
C27—C28—H28	120.00	C4—C5—H5	121.00
C32—C31—H31	121.00	C6—C5—H5	121.00
C30—C31—H31	121.00	C7—C8—H8	119.00
C31—C32—H32	120.00	C9—C8—H8	120.00
C33—C32—H32	120.00	C8—C9—H9	119.00
C32—C33—H33	120.00	C10—C9—H9	119.00
C34—C33—H33	120.00	C9—C10—H10	121.00
C35—C34—H34	120.00	C11—C10—H10	121.00
C33—C34—H34	120.00	C10—C11—H11	119.00
C35—C36—H36B	109.00	C12—C11—H11	119.00
C35—C36—H36A	109.00	C12—C13—H13A	110.00
H36A—C36—H36B	108.00	C12—C13—H13B	110.00
C37—C36—H36B	109.00	C14—C13—H13A	110.00
C37—C36—H36A	109.00	C14—C13—H13B	110.00
C40—C39—H39A	108.00	H13A—C13—H13B	108.00
H39A—C39—H39B	107.00	C15—C16—H16A	109.00
C38—C39—H39B	108.00	C15—C16—H16B	109.00
C40—C39—H39B	108.00	C17—C16—H16A	109.00
C38—C39—H39A	108.00	C17—C16—H16B	109.00
C39—C40—H40A	107.00	H16A—C16—H16B	108.00
C39—C40—H40B	107.00	C16—C17—H17A	109.00
H40A—C40—H40B	107.00	C16—C17—H17B	109.00
C41—C40—H40B	107.00	C18—C17—H17A	109.00
C41—C40—H40A	107.00	C18—C17—H17B	109.00
C44A—C41—H41	104.00	H17A—C17—H17B	108.00
C44B—C41—H41	50.00	C17—C18—H18	108.00
C42—C41—H41	104.00	C19—C18—H18	108.00
C40—C41—H41	104.00	C21—C18—H18	108.00
C43—C42—H42B	108.00	C18—C19—H19A	109.00
C41—C42—H42A	108.00	C18—C19—H19B	109.00
C41—C42—H42B	108.00	C20—C19—H19A	109.00
H42A—C42—H42B	107.00	C20—C19—H19B	109.00
C43—C42—H42A	108.00	H19A—C19—H19B	108.00
C38—C43—H43B	108.00	C15—C20—H20A	109.00
C38—C43—H43A	108.00	C15—C20—H20B	109.00
C42—C43—H43A	108.00	C19—C20—H20A	109.00
C42—C43—H43B	108.00	C19—C20—H20B	109.00
H43A—C43—H43B	107.00	H20A—C20—H20B	108.00
C45A—C44A—H44B	107.00	C18—C21—H21A	108.00
H44A—C44A—H44B	107.00	C18—C21—H21B	108.00
C41—C44A—H44B	107.00	C22—C21—H21A	108.00
C45A—C44A—H44A	107.00	C22—C21—H21B	108.00
C41—C44A—H44A	107.00	H21A—C21—H21B	107.00
C45B—C44B—H41	132.00	C21—C22—H22A	109.00
H41—C44B—H44C	119.00	C21—C22—H22B	109.00
C41—C44B—H44D	109.00	C23—C22—H22A	109.00
C45B—C44B—H44D	108.00	C23—C22—H22B	109.00
H44C—C44B—H44D	107.00	H22A—C22—H22B	108.00
C41—C44B—H44C	108.00	C22—C23—H23A	109.00
H41—C44B—H44D	70.00	C22—C23—H23B	110.00
C45B—C44B—H44C	108.00	C22—C23—H23C	109.00
H45A—C45A—H45B	109.00	H23A—C23—H23B	110.00
C46A—C45A—H45A	111.00	H23A—C23—H23C	109.00
C46A—C45A—H45B	111.00	H23B—C23—H23C	109.00
			
C24—N4—C30—C35	167.8 (4)	C38—C39—C40—C41	27.6 (14)
C30—N4—C24—C25	128.2 (4)	C39—C40—C41—C42	−36.2 (12)
C30—N4—C24—C29	−58.4 (6)	C39—C40—C41—C44A	−170.9 (11)
C24—N4—C30—C31	−13.1 (6)	C44A—C41—C42—C43	−177.3 (11)
C37—N5—N6—C38	−175.4 (5)	C40—C41—C42—C43	45.1 (11)
N6—N5—C37—O2	−1.1 (6)	C42—C41—C44A—C45A	−179.5 (12)
N6—N5—C37—C36	178.6 (4)	C40—C41—C44A—C45A	−47 (2)
N5—N6—C38—C43	−178.8 (6)	C41—C42—C43—C38	−46.4 (12)
N5—N6—C38—C39	1.3 (10)	C41—C44A—C45A—C46A	−62.9 (18)
C1—N1—C7—C8	−9.6 (7)	C2—C1—C6—Cl2	−179.1 (5)
C7—N1—C1—C6	115.8 (6)	N1—C1—C6—Cl2	0.1 (8)
C1—N1—C7—C12	171.5 (4)	N1—C1—C6—C5	−179.9 (7)
C7—N1—C1—C2	−65.1 (7)	N1—C1—C2—Cl1	−4.7 (8)
N3—N2—C14—O1	6.3 (6)	N1—C1—C2—C3	175.8 (6)
C14—N2—N3—C15	−174.7 (5)	C6—C1—C2—Cl1	174.5 (5)
N3—N2—C14—C13	−171.4 (4)	C6—C1—C2—C3	−5.0 (9)
N2—N3—C15—C20	3.9 (8)	C2—C1—C6—C5	0.9 (10)
N2—N3—C15—C16	−176.3 (5)	C1—C2—C3—C4	6.3 (12)
N4—C24—C25—Cl4	−4.0 (5)	Cl1—C2—C3—C4	−173.2 (7)
C25—C24—C29—C28	−4.2 (6)	C2—C3—C4—C5	−3.3 (14)
N4—C24—C29—Cl3	0.2 (6)	C3—C4—C5—C6	−0.8 (14)
N4—C24—C25—C26	175.9 (4)	C4—C5—C6—C1	2.0 (12)
C29—C24—C25—Cl4	−178.0 (3)	C4—C5—C6—Cl2	−178.1 (7)
C25—C24—C29—Cl3	174.0 (3)	C8—C7—C12—C11	−1.7 (6)
C29—C24—C25—C26	1.9 (6)	C8—C7—C12—C13	−179.9 (4)
N4—C24—C29—C28	−178.0 (4)	C12—C7—C8—C9	1.1 (7)
C24—C25—C26—C27	1.3 (8)	N1—C7—C12—C11	177.3 (4)
Cl4—C25—C26—C27	−178.9 (5)	N1—C7—C8—C9	−177.8 (5)
C25—C26—C27—C28	−2.3 (9)	N1—C7—C12—C13	−1.0 (6)
C26—C27—C28—C29	−0.1 (9)	C7—C8—C9—C10	0.2 (8)
C27—C28—C29—C24	3.4 (8)	C8—C9—C10—C11	−0.8 (9)
C27—C28—C29—Cl3	−174.8 (5)	C9—C10—C11—C12	0.2 (8)
N4—C30—C31—C32	−177.6 (5)	C10—C11—C12—C7	1.1 (7)
C31—C30—C35—C36	179.7 (4)	C10—C11—C12—C13	179.3 (4)
C35—C30—C31—C32	1.5 (7)	C11—C12—C13—C14	−100.4 (4)
N4—C30—C35—C34	178.7 (4)	C7—C12—C13—C14	77.9 (5)
N4—C30—C35—C36	−1.2 (6)	C12—C13—C14—O1	−75.5 (5)
C31—C30—C35—C34	−0.4 (7)	C12—C13—C14—N2	102.3 (4)
C30—C31—C32—C33	−2.2 (10)	N3—C15—C16—C17	−131.7 (6)
C31—C32—C33—C34	1.8 (12)	C20—C15—C16—C17	48.2 (9)
C32—C33—C34—C35	−0.6 (11)	N3—C15—C20—C19	133.7 (6)
C33—C34—C35—C36	179.8 (6)	C16—C15—C20—C19	−46.1 (8)
C33—C34—C35—C30	−0.1 (9)	C15—C16—C17—C18	−54.2 (9)
C30—C35—C36—C37	80.0 (6)	C16—C17—C18—C19	57.9 (8)
C34—C35—C36—C37	−100.0 (5)	C16—C17—C18—C21	−176.6 (7)
C35—C36—C37—O2	−61.9 (5)	C17—C18—C19—C20	−57.3 (9)
C35—C36—C37—N5	118.4 (4)	C21—C18—C19—C20	176.7 (7)
C39—C38—C43—C42	34.4 (11)	C17—C18—C21—C22	161.5 (8)
N6—C38—C43—C42	−145.6 (8)	C19—C18—C21—C22	−76.0 (11)
N6—C38—C39—C40	156.0 (8)	C18—C19—C20—C15	51.8 (9)
C43—C38—C39—C40	−23.9 (12)	C18—C21—C22—C23	−77.6 (11)

### Hydrogen-bond geometry (Å, °)

**Table:**

*D*—H···*A*	*D*—H	H···*A*	*D*···*A*	*D*—H···*A*
N1—H1···O1	0.86	2.28	2.886 (4)	128
N2—H2···O2^i^	0.86	2.24	3.077 (5)	165
N4—H4A···O2	0.86	2.32	2.921 (6)	127
N5—H5A···O1^ii^	0.86	2.21	3.034 (4)	161
C20—H20A···O2^i^	0.97	2.53	3.330 (6)	139
C36—H36B···O1^ii^	0.97	2.41	3.307 (5)	155

Symmetry codes: (i) *x*+1, −*y*+3/2, *z*+1/2; (ii) −*x*+1, −*y*+1, −*z*+1.

## References

[bb1] Allen, F. H., Kennard, O., Watson, D. G., Brammer, L., Orpen, A. G. & Taylor, R. (1987). *J. Chem. Soc. Perkin Trans. 2*, pp. S1–19.

[bb2] Altomare, A., Burla, M. C., Camalli, M., Cascarano, G. L., Giacovazzo, C., Guagliardi, A., Moliterni, A. G. G., Polidori, G. & Spagna, R. (1999). *J. Appl. Cryst.* **32**, 115–119.

[bb3] Cremer, D. & Pople, J. A. (1975). *J. Am. Chem. Soc.* **97**, 1354–1358.

[bb4] Farrugia, L. J. (1997). *J. Appl. Cryst.* **30**, 565.

[bb5] Farrugia, L. J. (1999). *J. Appl. Cryst.* **32**, 837–838.

[bb6] Gobec, S., Brozic, P. & Rizner, T. L. (2005). *Bioorg. Med. Chem. Lett.* **15**, 5170–5175. 10.1016/j.bmcl.2005.08.063 16183274

[bb7] Moser, P., Sallmann, A. & Wiesenberg, I. (1990). *J. Med. Chem.* **33**, 2358–2368. 10.1021/jm00171a008 2118185

[bb8] Sallmann, A. (1986). *Am. J. Chem. ***80** (Suppl. 4B), 29–33.

[bb9] Sheldrick, G. M. (2008). *Acta Cryst.* A**64**, 112–122. 10.1107/S0108767307043930 18156677

[bb10] Sriram, D., Yogeeswari, P. & Devakaram, R. V. (2006). *Bioorg. Med. Chem.* **14**, 3113–3118. 10.1016/j.bmc.2005.12.042 16412646

[bb11] Stoe & Cie (2002). *X-AREA* and *X-RED32* Stoe & Cie, Darmstadt, Germany.

[bb12] Wittine, K., Benci, K., Rajic, Z., Zorc, B., Kralj, M., Marjanovic, M., Pavelic, K., De Clercq, E., Andrei, G., Snoeck, R., Balsarini, J. & Mintas, M. (2009). *Eur. J. Med. Chem.* **44**, 143–151. 10.1016/j.ejmech.2008.03.037 18485540

[bb13] Zhang, J., Wang, J., Wu, H., He, Y., Zhu, G., Cui, X. & Tang, L. (2009). *Bioorg. Med. Chem. Lett.* **19**, 3324–3327. 10.1016/j.bmcl.2009.04.050 19423341

